# Supplementary material for: Dysregulation of Peripheral Blood Mononuclear Cells and Immune-Related Proteins during the Early Post-Operative Immune Response in Ovarian Cancer Patients
Source: Cancers (Basel). 2023 Dec 30;16(1):190. doi: 10.3390/cancers16010190 (PMC10778568; doi:10.3390/cancers16010190)
Supplement: Supplementary file 1 [file cancers-16-00190-s001.zip › cancers-2784684-supplementary File S1.pdf]

PVDF membranes

Patients 37, 42, 48, 50 and 53 blots densitometric scanning were used for Figure 4B (left).

Patient 50 blots were used as representative for Figure 4B (right).

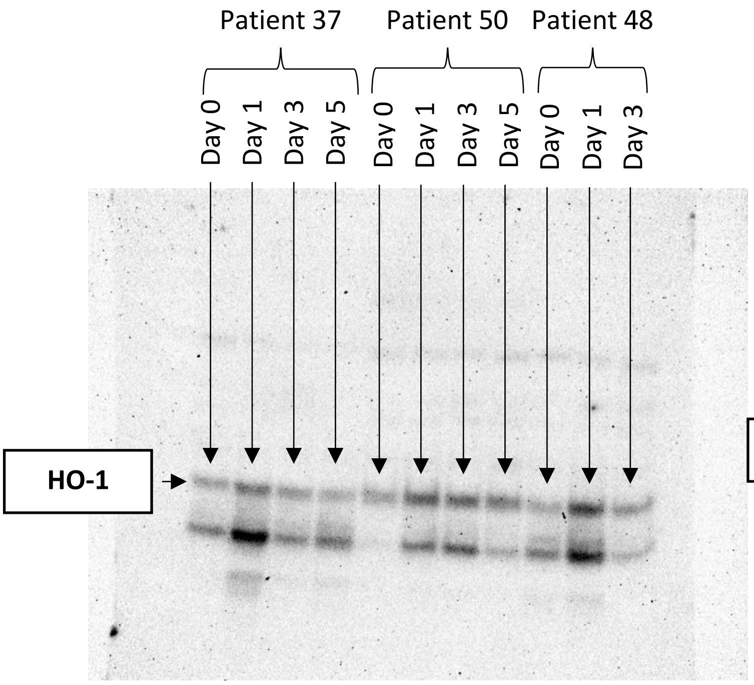

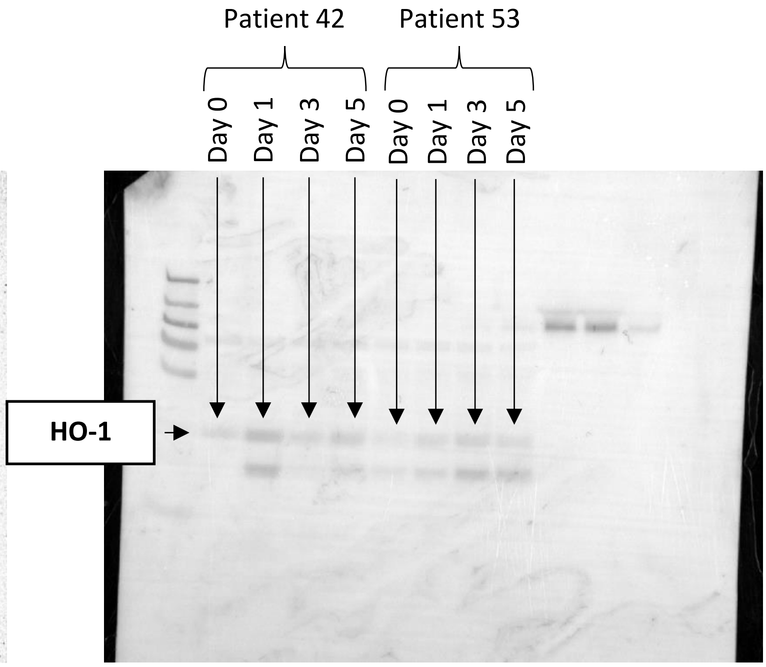

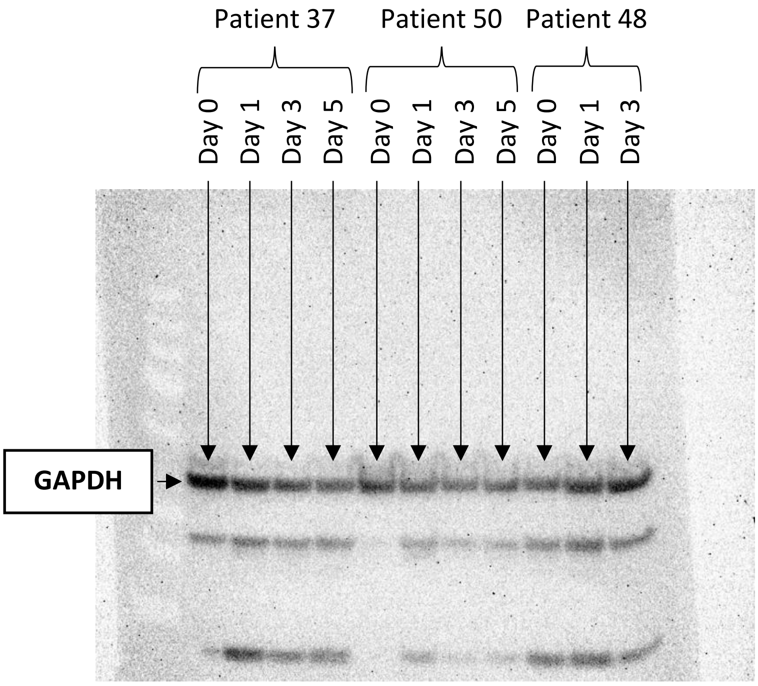

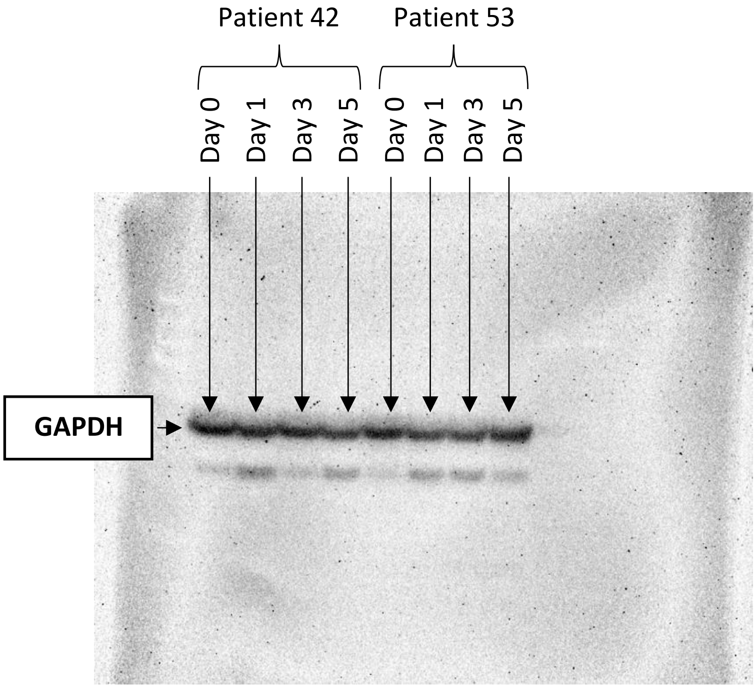

HO-1, heme oxygenase 1; GAPDH, glyceraldehyde-3-phosphate dehydrogenase.
